# Supplementary material for: A Comprehensive Analysis of FUT8 Overexpressing Prostate Cancer Cells Reveals the Role of EGFR in Castration Resistance
Source: Cancers (Basel). 2020 Feb 18;12(2):468. doi: 10.3390/cancers12020468 (PMC7072180; doi:10.3390/cancers12020468)
Supplement: Supplementary file 1 [file cancers-12-00468-s001.pdf]

cancers

Supplementary Materials: A Comprehensive Analysis of FUT8 Overexpressing Prostate Cancer Cells Reveals the Role of EGFR in Castration Resistance

Supplemental Figure 1

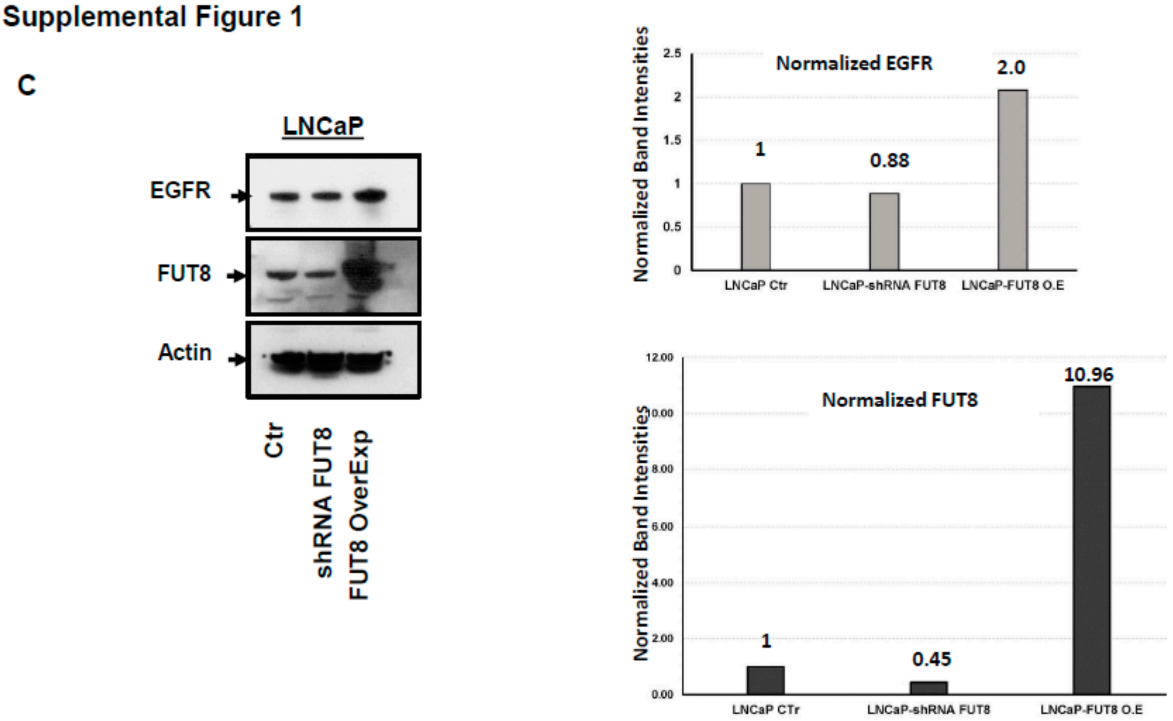

Figure S1. Western blot analysis for phosphorylated EGFR protein in LNCaP Ctr and LNCaP-FUT8 cells that were either mock treated (DMSO) or treated with EGF (2ng/ml). Actin is included to serve as equal amount of loading across the lanes (**A**). Differential expression of several phospho-peptides identified across cell lines when compare to the wildtype LNCaP-Ctr cells (**B**). Western blot analysis for the LNCaP Ctr and LNCaP-shRNA Fut8 cells showing downregulation of EGFR (**C**).

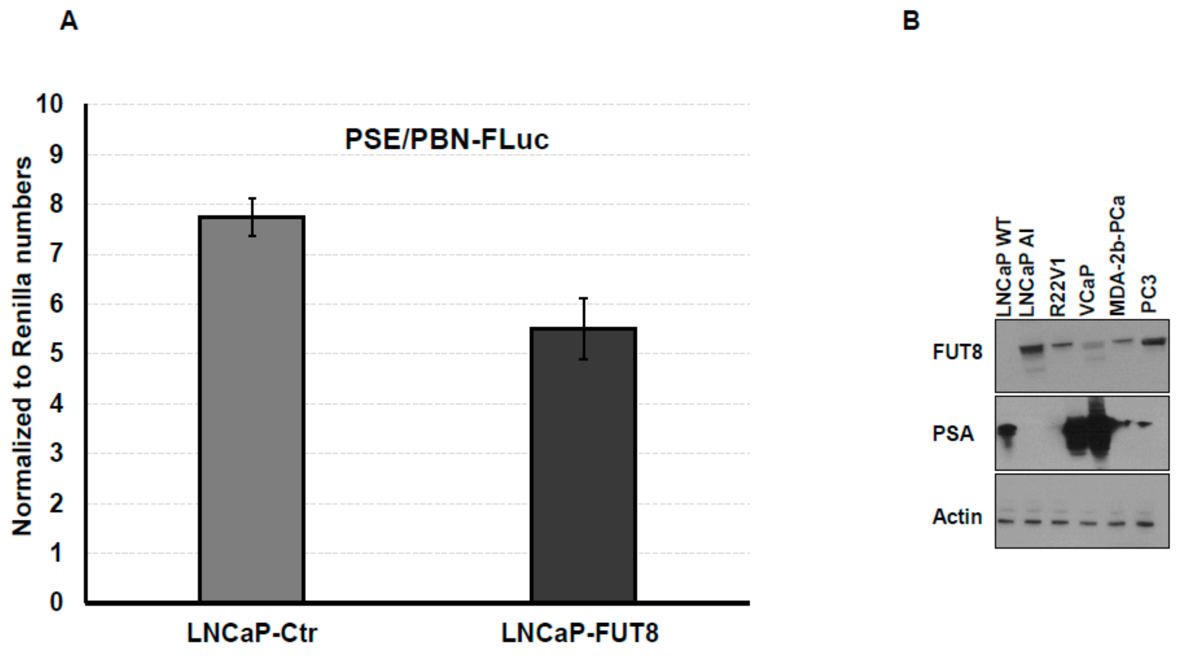

Figure S2. AR reporter assay showing the activity of PSE/PBN promoter in LNCaP Ctr and LNCaP-FUT8 cells (**A**) Western blot analysis for FUT8 and PSA in several different prostate cancer cell lines (**B**).

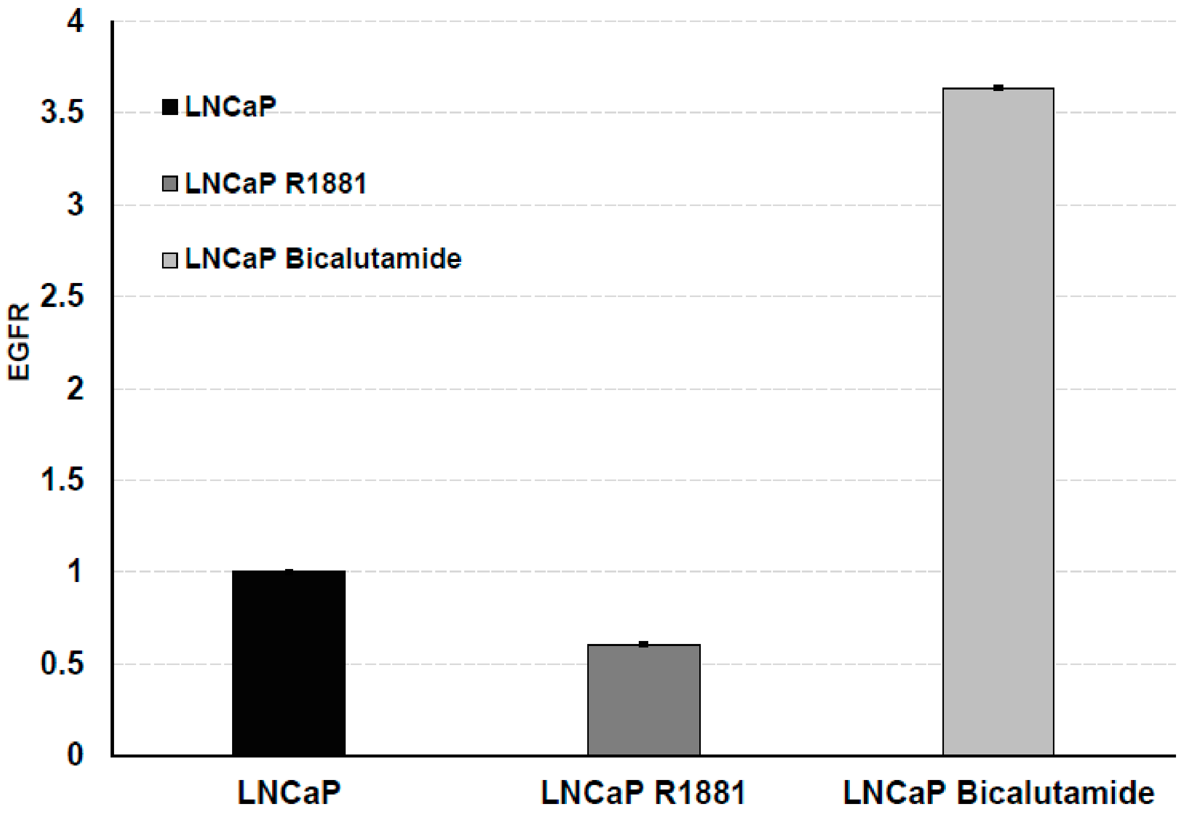

Figure S3. QRT PCR analysis showing relative expression of EGFR mRNA in cells that were treated with R1881 or with anti-androgen (Bicalutamide).

A Intensity ratio of each band Normalized to Actin Intensity

	LNCaP WT	LNCaP-FUT8
FUT8	0.446	0.863
EGFR	0.517	0.665
AR	0.757	0.801
PSA	1.116	0.847

Figure S4. Quantitation of western blot using ImageJ in LNCaP WT and LNCaP-FUT8 for figure 3A and B.

Supplemental Table 3a

Index	Label	log2(LNCaP-Fut8)	log2(PC3)	log2(LNCaP-95)	RowLabel	High_Man	Sialic	Fuc	Others	GlyLabel
DSISINATNIK_N2H10	N352_HexNAc(2)Hex(10)	2.281536508	1.745930216	1.544194624	EGFR	1	0	0	0	High_Man
DSISINATNIK_N2H4	N352_HexNAc(2)Hex(4)	0.157335282	0.863108056	1.191806893	EGFR	1	0	0	0	High_Man
DSISINATNIK_N2H5	N352_HexNAc(2)Hex(5)	0.396228836	0.397908839	0.534980489	EGFR	1	0	0	0	High_Man
DSISINATNIK_N2H5F1	N352_HexNAc(2)Hex(5)Fuc(1)	0.347006672	0.106117502	0.190493903	EGFR	0	0	1	0	Fucose
DSISINATNIK_N2H6	N352_HexNAc(2)Hex(6)	0.661104339	0.634133998	0.256901508	EGFR	1	0	0	0	High_Man
DSISINATNIK_N2H6F1	N352_HexNAc(2)Hex(6)Fuc(1)	2.033087438	2.271206919	0.266873208	EGFR	0	0	1	0	Fucose
DSISINATNIK_N2H7	N352_HexNAc(2)Hex(7)	2.377751257	2.845200045	0.587646452	EGFR	1	0	0	0	High_Man
DSISINATNIK_N2H7F1	N352_HexNAc(2)Hex(7)Fuc(1)	1.954272233	2.097150725	0.257012574	EGFR	0	0	1	0	Fucose
DSISINATNIK_N2H8	N352_HexNAc(2)Hex(8)	1.968219964	1.882512616	0.043141685	EGFR	1	0	0	0	High_Man
DSISINATNIK_N2H9	N352_HexNAc(2)Hex(9)	1.77972981	1.611446623	0.981005875	EGFR	1	0	0	0	High_Man
DSISINATNIK_N4H4S1	N352_HexNAc(4)Hex(4)NeuAc(1)	1.174165198	1.277168108	-0.286061636	EGFR	0	1	0	0	Sialic
DSISINATNIK_N4H4F1	N352_HexNAc(4)Hex(4)Fuc(1)	0.520727072	0.572157631	0.54298122	EGFR	0	0	1	0	Fucose
DSISINATNIK_N4H4F2	N352_HexNAc(4)Hex(4)Fuc(2)	1.493874327	1.72051809	0.174441385	EGFR	0	0	1	0	Fucose
DSISINATNIK_N6H4	N352_HexNAc(6)Hex(4)	1.591847009	1.636491822	0.909359565	EGFR	0	0	0	1	Others
DSISINATNIK_N6H5	N352_HexNAc(6)Hex(5)	1.562503669	1.021037045	0.953268895	EGFR	0	0	0	1	Others
NCTSISGDLHILPVAFR_N2H6	N361_HexNAc(2)Hex(6)	-0.089733346	-0.881571903	-0.546584611	EGFR	1	0	0	0	High_Man
NCTSISGDLHILPVAFR_N2H7	N361_HexNAc(2)Hex(7)	0.303394753	-0.524193275	-0.857775485	EGFR	1	0	0	0	High_Man
NCTSISGDLHILPVAFR_N2H8	N361_HexNAc(2)Hex(8)	0.029247539	-0.310308457	-0.148136411	EGFR	1	0	0	0	High_Man
NCTSISGDLHILPVAFR_N4H4F1	N361_HexNAc(4)Hex(4)Fuc(1)	0.268611573	-0.018577045	0.180085311	EGFR	0	0	1	0	Fucose
NCTSISGDLHILPVAFR_N4H5F1	N361_HexNAc(4)Hex(5)Fuc(1)	0.494577845	0.465247283	0.097712162	EGFR	0	0	1	0	Fucose
NCTSISGDLHILPVAFR_N4H6F1	N361_HexNAc(4)Hex(6)Fuc(1)	1.290264143	1.311640091	0.449364372	EGFR	0	0	1	0	Fucose
NCTSISGDLHILPVAFR_N4H7F1	N361_HexNAc(4)Hex(7)Fuc(1)	0.636479126	0.94183678	0.519462108	EGFR	0	0	1	0	Fucose
LLIQAWPENR_N4H5F1	N413_HexNAc(4)Hex(5)Fuc(1)	-0.02366056	-1.031971808	-0.605217975	EGFR	0	0	1	0	Fucose
LLIQAWPENR_N4H5F2	N413_HexNAc(4)Hex(5)Fuc(2)	0.24825543	-0.742616566	-0.085881182	EGFR	0	0	1	0	Fucose
TCPAGVMGENNTLVWK_N2H12	N603_HexNAc(2)Hex(12)	-0.126125358	0.163035408	1.21288094	EGFR	1	0	0	0	High_Man
TCPAGVMGENNTLVWK_N2H8	N603_HexNAc(2)Hex(8)	0.81621641	0.90815393	0.2203077	EGFR	1	0	0	0	High_Man
TCPAGVMGENNTLVWK_N4H5F1	N603_HexNAc(4)Hex(5)Fuc(1)	0.671891766	0.032315783	0.527900002	EGFR	0	0	1	0	Fucose
TCPAGVMGENNTLVWK_N4H5F1S1	N603_HexNAc(4)Hex(5)Fuc(1)NeuAc(1)	0.670303892	1.275141235	2.606762278	EGFR	0	1	1	0	Sialic
TCPAGVMGENNTLVWK_N4H5F3	N603_HexNAc(4)Hex(5)Fuc(3)	0.340046573	0.439915604	1.674638367	EGFR	0	0	1	0	Fucose
TCPAGVMGENNTLVWK_N6H7	N603_HexNAc(6)Hex(7)	-0.043013751	0.355739112	1.560981046	EGFR	0	0	0	1	Others
YADAGHVCHLCHPNCTYGCTGPGLEGCPTNGPK_N2H5	N623_HexNAc(2)Hex(5)	0.509694414	0.22595487	0.283675536	EGFR	1	0	0	0	High_Man
YADAGHVCHLCHPNCTYGCTGPGLEGCPTNGPK_N2H6	N623_HexNAc(2)Hex(6)	1.00998893	0.430777198	-0.302030653	EGFR	1	0	0	0	High_Man
YADAGHVCHLCHPNCTYGCTGPGLEGCPTNGPK_N3H5F1	N623_HexNAc(3)Hex(5)Fuc(1)	1.397379953	1.292584461	0.18147891	EGFR	0	0	1	0	Fucose
YADAGHVCHLCHPNCTYGCTGPGLEGCPTNGPK_N4H4F1	N623_HexNAc(4)Hex(4)Fuc(1)	0.708429231	0.297910351	-0.425135388	EGFR	0	0	1	0	Fucose

Supplemental Table 3b

Index	Label	log2(LNCaP-Fut8)	log2(PC3)	log2(LNCaP-95)	RowLabel	High_Man	Sialic	Fuc	Others	GlyLabel
DSISINATNIK_N2H10	N352_HexNAc(2)Hex(10)	2.281537	1.74583	1.544196	EGFR	1	0	0	0	High_Man
DSISINATNIK_N2H4	N352_HexNAc(2)Hex(4)	0.157336	0.883108	1.181807	EGFR	1	0	0	0	High_Man
DSISINATNIK_N2H5	N352_HexNAc(2)Hex(5)	0.386229	0.387909	0.63498	EGFR	1	0	0	0	High_Man
DSISINATNIK_N2H5F1	N352_HexNAc(2)Hex(5)Fuc(1)	0.347007	0.106118	0.190494	EGFR	0	0	1	0	Fucose
DSISINATNIK_N2H6	N352_HexNAc(2)Hex(6)	0.661104	0.634134	0.268802	EGFR	1	0	0	0	High_Man
DSISINATNIK_N2H6F1	N352_HexNAc(2)Hex(6)Fuc(1)	2.033087	2.271207	0.266873	EGFR	0	0	1	0	Fucose
DSISINATNIK_N2H7	N352_HexNAc(2)Hex(7)	2.377751	2.8452	0.587846	EGFR	1	0	0	0	High_Man
DSISINATNIK_N2H7F1	N352_HexNAc(2)Hex(7)Fuc(1)	1.954272	2.097151	0.257013	EGFR	0	0	1	0	Fucose
DSISINATNIK_N2H8	N352_HexNAc(2)Hex(8)	1.98822	1.882513	0.043142	EGFR	1	0	0	0	High_Man
DSISINATNIK_N2H9	N352_HexNAc(2)Hex(9)	1.77973	1.611447	0.881006	EGFR	1	0	0	0	High_Man
DSISINATNIK_N3H4F3	N352_HexNAc(3)Hex(4)Fuc(3)	0.696807	0.611492	0.20468	EGFR	0	0	1	0	Fucose
DSISINATNIK_N4H4S1	N352_HexNAc(4)Hex(4)NeuAc(1)	1.174166	1.277168	-0.28606	EGFR	0	1	0	0	Sialic
DSISINATNIK_N4H4F1	N352_HexNAc(4)Hex(4)Fuc(1)	0.520727	0.572158	0.542981	EGFR	0	0	1	0	Fucose
DSISINATNIK_N4H4F2	N352_HexNAc(4)Hex(4)Fuc(2)	1.493874	1.720518	0.174441	EGFR	0	0	1	0	Fucose
NCTSISGDLHILPVAFR_N2H6	N361_HexNAc(2)Hex(6)	-0.08973	-0.88157	-0.54658	EGFR	1	0	0	0	High_Man
NCTSISGDLHILPVAFR_N2H7	N361_HexNAc(2)Hex(7)	0.303386	-0.62418	-0.85778	EGFR	1	0	0	0	High_Man
NCTSISGDLHILPVAFR_N2H8	N361_HexNAc(2)Hex(8)	0.029248	-0.31031	-0.14814	EGFR	1	0	0	0	High_Man
NCTSISGDLHILPVAFR_N4H4F1	N361_HexNAc(4)Hex(4)Fuc(1)	0.268612	-0.01858	0.180085	EGFR	0	0	1	0	Fucose
NCTSISGDLHILPVAFR_N4H5F1	N361_HexNAc(4)Hex(5)Fuc(1)	0.494578	0.465247	0.097712	EGFR	0	0	1	0	Fucose
NCTSISGDLHILPVAFR_N4H6F1	N361_HexNAc(4)Hex(6)Fuc(1)	1.290264	1.31164	0.449364	EGFR	0	0	1	0	Fucose
NCTSISGDLHILPVAFR_N4H7F1	N361_HexNAc(4)Hex(7)Fuc(1)	0.636479	0.941837	0.519462	EGFR	0	0	1	0	Fucose
LLIQAWPENR_N4H5F1	N413_HexNAc(4)Hex(5)Fuc(1)	-0.02366	-1.03197	-0.60522	EGFR	0	0	1	0	Fucose
LLIQAWPENR_N4H5F2	N413_HexNAc(4)Hex(5)Fuc(2)	0.248255	-0.74262	-0.08588	EGFR	0	0	1	0	Fucose
TCPAGVMGENNTLVWK_N2H12	N603_HexNAc(2)Hex(12)	-0.12613	0.183036	1.212881	EGFR	1	0	0	0	High_Man
TCPAGVMGENNTLVWK_N2H8	N603_HexNAc(2)Hex(8)	0.818216	0.908154	0.220308	EGFR	1	0	0	0	High_Man
TCPAGVMGENNTLVWK_N4H5F1	N603_HexNAc(4)Hex(5)Fuc(1)	0.671892	0.032316	0.5279	EGFR	0	0	1	0	Fucose
TCPAGVMGENNTLVWK_N4H5F1S1	N603_HexNAc(4)Hex(5)Fuc(1)NeuAc(1)	0.670304	1.275141	2.606762	EGFR	0	1	1	0	Sialic
TCPAGVMGENNTLVWK_N4H5F3	N603_HexNAc(4)Hex(5)Fuc(3)	0.340047	0.439916	1.674638	EGFR	0	0	1	0	Fucose
YADAGHVCHLCHPNCTYGCTGPGLEGCPTNGPK_N2H5	N623_HexNAc(2)Hex(5)	0.509684	0.225956	0.283876	EGFR	1	0	0	0	High_Man
YADAGHVCHLCHPNCTYGCTGPGLEGCPTNGPK_N2H6	N623_HexNAc(2)Hex(6)	1.009988	0.430777	-0.30203	EGFR	1	0	0	0	High_Man
YADAGHVCHLCHPNCTYGCTGPGLEGCPTNGPK_N3H5F1	N623_HexNAc(3)Hex(5)Fuc(1)	1.39738	1.292584	0.181479	EGFR	0	0	1	0	Fucose
YADAGHVCHLCHPNCTYGCTGPGLEGCPTNGPK_N4H4F1	N623_HexNAc(4)Hex(4)Fuc(1)	0.708429	0.29791	-0.42514	EGFR	0	0	1	0	Fucose

Supplemental Table 3c

Index	Label	log2(LNCaP-Fut8)	log2(PC3)	log2(LNCaP-95)	RowLabel	High_Man	Sialic	Fuc	Others	GlyLabel
DSISINATNIK_N2H5F1	N352_HexNAc(2)Hex(5)Fuc(1)	0.347007	0.106118	0.190494	EGFR	0	0	1	0	Fucose
DSISINATNIK_N2H6F1	N352_HexNAc(2)Hex(6)Fuc(1)	2.033087	2.271207	0.266873	EGFR	0	0	1	0	Fucose
DSISINATNIK_N2H7F1	N352_HexNAc(2)Hex(7)Fuc(1)	1.954272	2.097151	0.257013	EGFR	0	0	1	0	Fucose
DSISINATNIK_N4H4F1	N352_HexNAc(4)Hex(4)Fuc(1)	0.520727	0.572158	0.542981	EGFR	0	0	1	0	Fucose
DSISINATNIK_N4H4F2	N352_HexNAc(4)Hex(4)Fuc(2)	1.493874	1.720518	0.174441	EGFR	0	0	1	0	Fucose
NCTSISGDLHILPVAFR_N4H4F1	N361_HexNAc(4)Hex(4)Fuc(1)	0.268612	-0.01858	0.180085	EGFR	0	0	1	0	Fucose
NCTSISGDLHILPVAFR_N4H5F1	N361_HexNAc(4)Hex(5)Fuc(1)	0.494578	0.465247	0.097712	EGFR	0	0	1	0	Fucose
NCTSISGDLHILPVAFR_N4H6F1	N361_HexNAc(4)Hex(6)Fuc(1)	1.290264	1.31164	0.449364	EGFR	0	0	1	0	Fucose
NCTSISGDLHILPVAFR_N4H7F1	N361_HexNAc(4)Hex(7)Fuc(1)	0.636479	0.941837	0.519462	EGFR	0	0	1	0	Fucose
LLIQAWPENR_N4H5F1	N413_HexNAc(4)Hex(5)Fuc(1)	-0.02366	-1.03197	-0.60522	EGFR	0	0	1	0	Fucose
LLIQAWPENR_N4H5F2	N413_HexNAc(4)Hex(5)Fuc(2)	0.248255	-0.74262	-0.08588	EGFR	0	0	1	0	Fucose
TCPAGVMGENNTLVWK_N4H5F1	N603_HexNAc(4)Hex(5)Fuc(1)	0.671892	0.032316	0.5279	EGFR	0	0	1	0	Fucose
TCPAGVMGENNTLVWK_N4H5F1S1	N603_HexNAc(4)Hex(5)Fuc(1)NeuAc(1)	0.670304	1.275141	2.606762	EGFR	0	1	1	0	Sialic
TCPAGVMGENNTLVWK_N4H5F3	N603_HexNAc(4)Hex(5)Fuc(3)	0.340047	0.439916	1.674638	EGFR	0	0	1	0	Fucose
YADAGHVCHLCHPNCTYGCTGPGLEGCPTNGPK_N3H5F1	N623_HexNAc(3)Hex(5)Fuc(1)	1.39738	1.292584	0.181479	EGFR	0	0	1	0	Fucose
YADAGHVCHLCHPNCTYGCTGPGLEGCPTNGPK_N4H4F1	N623_HexNAc(4)Hex(4)Fuc(1)	0.708429	0.29791	-0.42514	EGFR	0	0	1	0	Fucose

Supplemental Table 4a

UniProt	Gene Symbol	Protein Name
Q15054	POLD3	POLD3; DNA polymerase delta 3, accessory subunit
P39748	FEN1	FEN1; flap structure-specific endonuclease 1
P18858	LIG1	LIG1; DNA ligase 1
P49736	MCM2	MCM2; minichromosome maintenance complex component 2
P25205	MCM3	MCM3; minichromosome maintenance complex component 3
P33991	MCM4	MCM4; minichromosome maintenance complex component 4
P33992	MCM5	MCM5; minichromosome maintenance complex component 5
Q14566	MCM6	MCM6; minichromosome maintenance complex component 6
P33993	MCM7	MCM7; minichromosome maintenance complex component 7
P12004	PCNA	PCNA; proliferating cell nuclear antigen
Q9NRF9	POLE3	7 POLE3; DNA polymerase epsilon 3, accessory subunit
P09884	POLA1	POLA1; DNA polymerase alpha 1, catalytic subunit
P49005	POLD2	POLD2; DNA polymerase delta 2, accessory subunit
P49642	PRIM1	PRIM1; DNA primase subunit 1
P49643	PRIM2	PRIM2; DNA primase subunit 2
Q9NR33	POLE4	5 POLE4; DNA polymerase epsilon 4, accessory subunit
P35251	RFC1	RFC1; replication factor C subunit 1
P35250	RFC2	RFC2; replication factor C subunit 2
P40938	RFC3	RFC3; replication factor C subunit 3
P35249	RFC4	RFC4; replication factor C subunit 4
P40937	RFC5	RFC5; replication factor C subunit 5

Supplemental Table 4b

UniProt	Gene Symbol	Protein Name
P24941	CDK2	cyclin dependent kinase 2
Q9UM13	ANAPC10	anaphase promoting complex subunit 10
O14757	CHEK1	checkpoint kinase 1
Q9Y5N6	ORC6	origin recognition complex subunit 6
Q13257	MAD2L1	mitotic arrest deficient 2 like 1
P49736	MCM2	minichromosome maintenance complex component 2
P25205	MCM3	minichromosome maintenance complex component 3
P33991	MCM4	minichromosome maintenance complex component 4
P33992	MCM5	minichromosome maintenance complex component 5
Q14566	MCM6	minichromosome maintenance complex component 6
P33993	MCM7	minichromosome maintenance complex component 7
P12004	PCNA	proliferating cell nuclear antigen
P53350	PLK1	polo like kinase 1
Q13535	ATR	ATR serine/threonine kinase
P06400	RB1	RB transcriptional corepressor 1
P24385	CCND1	cyclin D1
O60566	BUB1B	BUB1 mitotic checkpoint serine/threonine kinase B
P33981	TTK	TTK protein kinase
O00311	CDC7	cell division cycle 7
P20248	CCNA2	cyclin A2
P14635	CCNB1	cyclin B1
Q99640	PKMYT1	protein kinase, membrane associated tyrosine/threonine 1
O95067	CCNB2	cyclin B2
O95997	PTTG1	PTTG1 regulator of sister chromatid separation, securin
P06493	CDK1	cyclin dependent kinase 1
Q12834	CDC20	cell division cycle 20
P30307	CDC25C	cell division cycle 25C
P30260	CDC27	cell division cycle 27
